# Strict and weak ordinal relations for estimating the criteria weights in Ordinal Priority Approach (OPA)

**DOI:** 10.1016/j.mex.2023.102389

**Published:** 2023-09-21

**Authors:** Amin Mahmoudi, Saad Ahmed Javed

**Affiliations:** a Department of Construction and Real Estate, School of Civil Engineering, Southeast University, 210096 Nanjing, China; b School of Business, Nanjing University of Information Science and Technology, 210044 Nanjing, China

**Keywords:** Ordinal Priority Approach, Optimization of criteria weights, Multi-criteria decision-making, Weak and strict preferences, Strict and Weak Ordinal Priority Approach

## Abstract

The Ordinal Priority Approach (OPA) is a recent multiple attribute decision-making that was published in 2020. This method uses a linear programming approach to solve decision-making problems in real-life situations. Due to its application in real-world situations, it has been employed by scholars widely in recent years. This study proposes two forms of the OPA for multiple attribute decision-making; one with strict ordinal relations and the other with weak ordinal relations. New forms are crucial in understanding the mathematical theory behind the original OPA. The study shows that one of the proposed forms and the original OPA are two forms of the same model. The study proves that the OPA is strict rather than weak. The study also found some new properties of the OPA. The application of the strict and weak OPA models is presented in a consumer modeling problem.

• Revisits the Ordinal Priority Approach (OPA) to Multiple Attribute Decision Making.
• Proposes the OPA with Strict Ordinal Relations (OPA-S) and Weak Ordinal Relations (OPA-W).
• Proposes a model to consider both weak and strict ordinal relations at the same time.

## Graphical abstract

**Figure:**
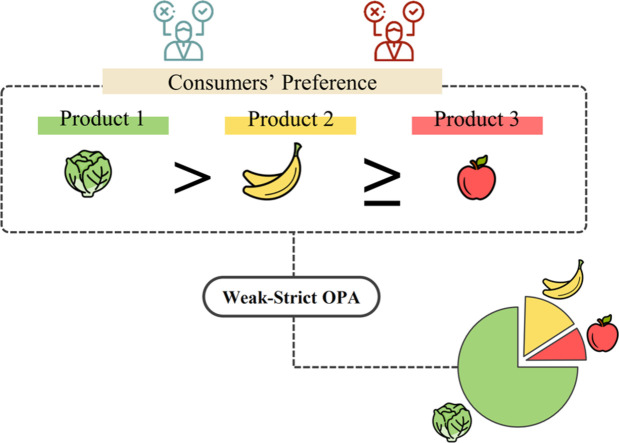

Specifications table

**Table:** 

Subject area:	Economics and Finance
More specific subject area:	*Multiple Criteria Decision-Making*
Name of your method:	*Strict and Weak Ordinal Priority Approach*
Name and reference of original method:	Ordinal Priority Approach (OPA) Ataei, Y., Mahmoudi, A., Feylizadeh, M. R., & Li, D. F. (2020). Ordinal priority approach (OPA) in multiple attribute decision-making. Applied Soft Computing, 86, 105893. 10.1016/j.asoc.2019.105893
Resource availability:	https://ordinalpriorityapproach.com

**Method details**

## Introduction

The Ordinal Priority Approach (OPA) is a new methodology for handling the multiple attribute decision-making problems where the decision-makers' preferences are defined through ordered relations rather than cardinal numbers [1]. Studies have confirmed the reliability of such an approach as it minimizes the information loss because it is easier to judge which alternative is better than the other instead of assigning cardinal scores to the alternatives [2]. Ordinal data is more useful than cardinal data for non-numerical representation of uncertainty [3]. Since its inception, the OPA and its extensions have been used to solve problems in different fields, e.g., blockchain technologies [4], green energies [5,6], supplier performance measurement [7], transport service quality evaluation [8], among others.

The OPA is a linear programming-driven mathematical approach where the input data are in the form of ordinal relations (preferences). Though the reliability of the approach has been verified empirically on multiple occasions, what kind of ordered relations the OPA supports has never been defined. The current study intends to fill this gap. Based on the classical theory of ordinal relations [9,10], first, the study defines the types of ordinal relations as weak and strict ordinal relations, and later by relying on this new conceptualization, it builds the OPA with weak and strict ordinal relations. Also, the latter's relationship with the original OPA is proven. The study is important to understand the mathematical theory of the Ordinal Priority Approach (OPA) for multiple attribute decision-making.

## Preliminaries

Imagine a decision-maker (DM) who faces the problem of choosing one attribute (or alternative) from a set of attributes (or alternatives). Let's denote this set by C*,* and $c_{1}$, $c_{2}$, … being the members of this set.

Definition 1 ***Ordinal relations*** [10]

Ordinal relation (also called order relation or preference relation) is asymmetric relation, i.e., there is no case where $c_{1}>c_{2}$ and $c_{1}<c_{2}$ are simultaneously possible. Thus, either $c_{1}\geq c_{2}$ or $c_{1}\leq c_{2}$.

Definition 2 ***Strict ordinal relations***[9,10]

A strict ordinal relation (>) is an ordinal relation that results in a strict linear ranking as determined by the weights (a ranking in which ties are not permissible), i.e., $c_{1}>c_{2}$ or $c_{1}<c_{2}$. More precisely, a strict ordinal relation follows the following properties:

- Irreflexivity: for no c is c<c,
- Transitivity: if $c_{1}>c_{2}$ and $c_{2}>c_{3}$, then $c_{1}>c_{3}$
- Acyclicity: if $c_{1}>c_{2}>c_{3}>\ldots >\;c_{n-1}>c_{n},$ then if $c_{n}\neq c_{1}$,

In short, the strong ordinal relation implies:$\;c_{1}$ is *strongly preferred* to $c_{2}$ (or vice versa).

Definition 3 ***Weak ordinal relations***[9,10]

Strict ordinal relation where *indifference* is permissible is called weak ordinal relation (≥), whereas indifference is the absence of strict preference in either direction, i.e., $c_{1}\geq c_{2}$ or $c_{1}\leq c_{2}$. Thus, a weak ordinal relation is an ordinal relation that results in a weak linear ranking as determined by the weights (a ranking in which ties are allowed). In short, the weak ordinal relation encompasses two relations;$\;c_{1}$ is *weakly preferred* to $c_{2}$ (or vice versa) and $c_{1}$ is *indifferent* to $c_{2}$.

## Ordinal Priority Approach with Weak Ordinal Relations (OPA-W)

In this section, the OPA-W will be proposed. Consider the sets, parameters and variables as defined in Table 1.

**Table 1:** The sets, indexes, variables and other parameters of the proposed models.

**Set**	
C	Set of attributes $\left\{c_{1},\;c_{2},\;\ldots ,\;c_{n}\right\}$
**Indexes**	
j	Index of the attributes (j=1,2,…,n)
r	Rank of the attributes (r=1,2,…,n)
**Variables**	
$c_{j}^{\left(r\right)}$	Attribute j with rank r in the attributes set
$W_{j}^{\left(r\right)}$	Weight of attribute j with rank r in the attributes set

Assume that there is a weak ordinal relation among the attributes (or alternatives) (see Definition 3), i.e.,

(1) $$c_{j^{\prime }}^{\left(1\right)}\geq c_{j^{\prime \prime }}^{\left(2\right)}\geq \cdots \geq c_{j^{\prime \prime \prime }}^{\left(n-1\right)}\geq c_{j^{\prime \prime \prime }}^{\left(n\right)}$$

where the subscripts are just different indexes of the attributes; however, since we do not know their exact index, for the sake of convenience, we can choose to represent them generally by $\{j^{\prime },j^{\prime \prime },\ldots \}\in j$.

The weights of these attributes are expected to have the same ordered relation. Thus,

(2) $$W_{j^{\prime }}^{\left(1\right)}\geq W_{j^{\prime \prime }}^{\left(2\right)}\geq \ldots \geq W_{j^{\prime \prime \prime }}^{\left(n-1\right)}\geq W_{j^{\prime \prime \prime }{}^{\prime }}^{\left(n\right)}$$

According to the definition of weak ordinal relations (Definition 3), an attribute can be *indifferent* to the other attribute and can be *weakly preferred* to the other attribute as well; thus, the difference between the weights of two attributes can either be zero or slightly more than zero. Therefore, one gets the following relations,

(3) $$\begin{array}{c} \begin{array}{c} W_{j^{\prime }}^{\left(1\right)}-W_{j^{\prime \prime }}^{\left(2\right)}\geq 0\\ \vdots \end{array}\\ W_{j^{\prime \prime \prime }}^{\left(n-1\right)}-W_{j^{\prime \prime \prime }{}^{\prime }}^{\left(n\right)}\geq 0 \end{array}$$

In general, Eq. (3) can be expressed as,

(4) $$W_{j^{\prime }}^{\left(r\right)}-W_{j^{\prime \prime }}^{\left(r+1\right)}\geq 0\;r=1\cdots n$$

With the aim of maximizing the preference of the attributes, the maximization of Eq. (4) can produce an objective function, presented in Eq. (5), and thus, the values of weights of the attributes can be calculated:

(5) $$\begin{array}{cc} Max\;\left\{\left(W_{j^{\prime }}^{\left(r\right)}-W_{j^{\prime \prime }}^{\left(r+1\right)}\right),\;\left(W_{j^{\prime \prime \prime }{}^{\prime }}^{\left(n\right)}\right)\right\} & r=1\ldots n\\ \sum_{j=1}^{n}W_{j}^{\left(r\right)}=1\\ W_{j}^{\left(r\right)}\geq 0 & \forall \;j\;\left(j=1\ldots n\right) \end{array}$$

Eq. (5) represents a multi-objective model, which can be transformed into a single-objective model using the Max-Min approach (maximization of the minimization objectives) [11]. If we denote the minimization of the objective function by $Z^{\prime }$, the resultant single-objective model is the OPA-W given by:

(6) $$\begin{array}{cc} Max\;Z^{\prime }\\ W_{j^{\prime }}^{\left(r\right)}-W_{j^{\prime \prime }}^{\left(r+1\right)}\geq Z^{\prime } & \forall \;r\;\left(r=1\ldots n-1\right)\\ W_{j^{\prime \prime \prime }}^{\left(n\right)}\geq Z^{\prime } & r=n\\ \sum_{j=1}^{n}W_{j}^{\left(r\right)}=1\\ W_{j}^{\left(r\right)}\geq 0 & \forall \;j\;\left(j=1\ldots n\right) \end{array}$$

## Ordinal Priority Approach with Strong Ordinal Relations (OPA-S)

In this section, the OPA-S will be proposed. Consider the sets, parameters, and variables as defined in Table 1. Assume that there is a strict ordinal relation among the attributes (or alternatives) (see Definition 2), i.e.,

(7) $$c_{j^{\prime }}^{\left(1\right)}>c_{j^{\prime \prime }}^{\left(2\right)}>\cdots >c_{j^{\prime \prime \prime }}^{\left(n-1\right)}>c_{j^{\prime \prime \prime \prime }}^{\left(n\right)}$$

The weights of these attributes are expected to have the same ordinal relation. Thus,

(8) $$W_{j^{\prime }}^{\left(1\right)}>W_{j^{\prime \prime }}^{\left(2\right)}>\ldots >W_{j^{\prime \prime \prime }}^{\left(n-1\right)}>W_{j^{\prime \prime \prime }{}^{\prime }}^{\left(n\right)}$$

According to the definition of strict ordinal relations (Definition 2), an attribute can be *strongly preferred* to the other attribute; thus, the difference between the weights of two attributes is always more than zero. Let's assume a constant $\frac{1}{n}>0$, where n is the total number of attributes. Therefore, if the difference among the weights of the attributes is equal (i.e., the attributes are ordinally equidistant), one gets the following relation,

(9) $$\begin{array}{c} \begin{array}{c} W_{j^{\prime }}^{\left(1\right)}-W_{j^{\prime \prime }}^{\left(2\right)}=\frac{1}{n}\\ \vdots \end{array}\\ W_{j^{\prime \prime \prime }}^{\left(n-1\right)}-W_{j^{\prime \prime \prime }{}^{\prime }}^{\left(n\right)}=\frac{1}{n} \end{array}$$

To consider the impact of the ranks of attributes on the difference among the attributes, the left-hand side of Eq. (9) can be multiplied with r≥1, i.e.,

(10) $$r\;\left(W_{j^{\prime }}^{\left(r\right)}-W_{j^{\prime \prime }}^{\left(r+1\right)}\right)\geq \frac{1}{n}$$

It can be rewritten as:

(11) $$\left(W_{j^{\prime }}^{\left(r\right)}-W_{j^{\prime \prime }}^{\left(r+1\right)}\right)-\frac{1}{rn}\geq 0$$

With the aim of maximizing the preference of the attributes, the maximization of Eq. (11) can produce an objective function, presented in Eq. (12), and thus, the values of weights of the attributes can be calculated:

(12) $$\begin{array}{cc} Max\;\left\{\left(W_{j^{\prime }}^{\left(r\right)}-W_{j^{\prime \prime }}^{\left(r+1\right)}\right)-\frac{1}{rn},\;\left(W_{j^{\prime \prime \prime }{}^{\prime }}^{\left(n\right)}\right)-\frac{1}{n^{2}}\right\} & r=1\ldots n\\ \sum_{j=1}^{n}W_{j}^{\left(r\right)}=1\\ W_{j}^{\left(r\right)}\geq 0 & \forall \;j\;\left(j=1\ldots n\right) \end{array}$$

Eq. (12) represents a multi-objective model, which can be transformed into a single-objective model using the Max-Min approach (maximization of the minimization objectives) [11]. If we represent the minimization of the objective function by $Z^{\prime }$, the resultant single-objective model is the OPA-S given by

(13) $$\begin{array}{cc} Max\;Z^{\prime }\\ \left(W_{j^{\prime }}^{\left(r\right)}-W_{j^{\prime \prime }}^{\left(r+1\right)}\right)-\frac{1}{rn}\geq Z^{\prime } & \forall \;r\;\left(r=1\ldots n-1\right)\\ \left(W_{j^{\prime \prime \prime }{}^{\prime }}^{\left(n\right)}\right)-\frac{1}{n^{2}}\geq Z^{\prime } & r=n\\ \sum_{j=1}^{n}W_{j}^{\left(r\right)}=1\\ W_{j}^{\left(r\right)}\geq 0 & \forall \;j\;\left(j=1\ldots n\right) \end{array}$$

## Relationship between the OPA and OPA-S

At this stage, a natural question may arise in the readers' minds: the original OPA follows strict ordinal relations or weak ordinal relations? This is the question largely left unanswered in the literature. Theorem 1 proves that the OPA and the OPA-S are two forms of the same model, and therefore, the original OPA follows strict ordinal relations.

Theorem 1 The weights of the OPA-S (as presented in Eq. (13)) are *equivalent* to those obtained through the original OPA (as proposed by [1]).

**Proof.** Consider the OPA-S model given by Eq. (13). Multiply the constraints in Eq. (13) by r≥1 to get Eq. (14).

(14) $$\begin{array}{cc} Max\;Z^{\prime }\\ r\left(W_{j^{\prime }}^{\left(r\right)}-W_{j^{\prime \prime }}^{\left(r+1\right)}\right)-\frac{1}{n}\geq rZ^{\prime } & \forall \;r\;\left(r=1\ldots n-1\right)\\ n\left(W_{j^{\prime \prime \prime }{}^{\prime }}^{\left(n\right)}\right)-\frac{1}{n}\geq nZ^{\prime } & r=n\\ \sum_{j=1}^{n}W_{j}^{\left(r\right)}=1\\ W_{j}^{\left(r\right)}\geq 0 & \forall \;j\;\left(j=1\ldots n\right) \end{array}$$

It should be noted that when r=1, the value $\left(W_{j^{\prime }}^{\left(r\right)}-W_{j^{\prime \prime }}^{\left(r+1\right)}\right)-\frac{1}{n}$ equals zero. Thus, we get $Z^{\prime }\leq 0$ as the first constraint consistently, and since it's a maximization function the maximum value of $Z^{\prime }$ is always 0. On the one hand, the maximizing objective function would not let $Z^{\prime }$ become negative and on the other hand, even if $Z^{\prime }$ gets a positive value, the model with the constraint $Z^{\prime }\leq 0$ will not let it become positive. This constraint is important in determining the feasible region. The first constraint in the model (i.e., $Z^{\prime }=0$) makes other constraints redundant in determining the optimum value of $Z^{\prime }$. Therefore, replacing $rZ^{\prime }$ with $Z^{\prime }$ will not make any difference except bringing convenience in the modeling. Now, Eq. (14) can be rewritten as,

(15) $$\begin{array}{cc} \mathrm{Max}Z^{\prime }\\ r\left(W_{j^{\prime }}^{\left(r\right)}-W_{j^{\prime \prime }}^{\left(r+1\right)}\right)-\frac{1}{n}\geq Z^{\prime } & \forall \;r\;\left(r=1\cdots n-1\right)\\ n\left(W_{j^{\prime \prime \prime \prime }}^{\left(n\right)}\right)-\frac{1}{n}\geq Z^{\prime } & r=n\\ \sum_{j=1}^{n}W_{j}^{\left(r\right)}=1\\ W_{j}^{\left(r\right)}\geq 0 & \forall \;j\;\left(j=1\cdots n\right) \end{array}$$

The model in Eq. (15) can be rewritten as,

(16) $$\begin{array}{cc} Max\;Z^{\prime }\\ r\left(W_{j^{\prime }}^{\left(r\right)}-W_{j^{\prime \prime }}^{\left(r+1\right)}\right)\geq Z^{\prime }+\frac{1}{n} & \forall \;r\;\left(r=1\ldots n-1\right)\\ n\left(W_{j^{\prime \prime \prime }{}^{\prime }}^{\left(n\right)}\right)\geq Z^{\prime }+\frac{1}{n} & r=n\\ \sum_{j=1}^{n}W_{j}^{\left(r\right)}=1\\ W_{j}^{\left(r\right)}\geq 0 & \forall \;j\;\left(j=1\ldots n\right) \end{array}$$

Let $Z=Z^{\prime }+\frac{1}{n}$ and Eq. (16) becomes,

(17) $$\begin{array}{cc} Max\;Z-\frac{1}{n}\\ r\left(W_{j^{\prime }}^{\left(r\right)}-W_{j^{\prime \prime }}^{\left(r+1\right)}\right)\geq Z & \forall \;r\;\left(r=1\ldots n-1\right)\\ n\left(W_{j^{\prime \prime \prime }{}^{\prime }}^{\left(n\right)}\right)\geq Z & r=n\\ \sum_{j=1}^{n}W_{j}^{\left(r\right)}=1\\ W_{j}^{\left(r\right)}\geq 0 & \forall \;j\;\left(j=1\ldots n\right) \end{array}$$

Eq. (17) represents the OPA model of Ataei et al. [1]. Hence, Theorem 1 is proved. This revelation is no small contribution to the theory of multiple attribute decision-making, in general, and the Ordinal Priority Approach, in particular, for some reasons. For instance, Theorem 1 shows that the two models produce comparable weights of the attributes despite having different objective functions. The fact that two procedures yield a comparable set of weights validates the logic on which the OPA is built. To the best of our knowledge, there is no other MADM technique that exhibits this kind of characteristic. Since the objective function in the OPA-S is always zero (see Theorem 2), the theorem also reveals the difference value of the weights $\left(W_{j^{\prime }}^{\left(r\right)}-W_{j^{\prime \prime }}^{\left(r+1\right)}\right)$ in the OPA that is always a constant ($\frac{1}{rn}$) thus allowing us to quantify the *ordinal distance* between the weights of the attributes following strict ordinal relations. Two other theorems directly flow from Theorem 1.

Theorem 2 The objective function of the OPA-S is always zero.

**Proof.** The derivation of Eq. (15) from Eq. (14) proves it.

Theorem 3 The OPA is more robust than the OPA-S.

**Proof.** It is proven from the literature [12] that the OPA model with a higher value objective function is more robust. Also, it is obvious from Theorem 1 that the objective function in the OPA has a higher value (positive value) than that of the OPA-S, whose objective function always equals zero. Therefore, the OPA is more robust than the OPA-S.

## Hybrid Weak-Strict Model

In this section, we consider a situation inducing both strict and weak ordinal relations at the same time. In this regard, it is essential to introduce two sets. Set s for the criteria which have strict ordinal relation with each other and set w for the criteria that have weak relation. Considering two defined sets and Models (6) and (13), Model (18) can be presented for situations that have both weak and strict ordinal relations.

(18) $$\begin{array}{ccc} \mathrm{Max}\;Z^{\prime }\\ \left(W_{j^{\prime }}^{\left(r\right)}-W_{j^{\prime \prime }}^{\left(r+1\right)}\right)-\frac{1}{\mathrm{rn}}\geq Z^{\prime } & \forall \;r\;\left(r=1\cdots n-1\right) & c_{j^{\prime }}^{\left(r\right)}\;\mathrm{and}\;c_{j^{\prime \prime }}^{\left(r+1\right)}\;\in s\\ W_{j^{\prime }}^{\left(r\right)}-W_{j^{\prime \prime }}^{\left(r+1\right)}\geq Z^{\prime } & \forall \;r\;\left(r=1\cdots n-1\right) & c_{j^{\prime }}^{\left(r\right)}\;\mathrm{and}\;c_{j^{\prime \prime }}^{\left(r+1\right)}\;\in w\\ \left(W_{j^{\prime \prime \prime \prime }}^{\left(n\right)}\right)-\frac{1}{n^{2}}\geq Z^{\prime } & r=n & c_{j^{\prime \prime \prime \prime }}^{\left(n\right)}\;\in s\\ W_{j^{\prime \prime \prime \prime }}^{\left(n\right)}\geq Z^{\prime } & r=n & c_{j^{\prime \prime \prime \prime }}^{\left(n\right)}\;\in w\\ \sum_{j=1}^{n}W_{j}^{\left(r\right)}=1 & & c_{j}^{\left(r\right)}\in w\;\mathrm{and}\;s\\ W_{j}^{\left(r\right)}\geq 0 & \forall \;j\;\left(j=1\cdots n\right) & c_{j}^{\left(r\right)}\in w\;\mathrm{and}\;s \end{array}$$

## Application

Assume that there are three consumers, and we aim to model their choices based on the ordinal relations. Also, there are three choices for the consumers, Product A, Product B, and Product C. Each consumer replied as follows:

Consumer 1: Product B is *in fact better* than Product A, and Product A is *in fact better* than Product C.
Consumer 2: Product B is better (or *almost better)* than Product A, and Product A is better (or *almost better)* than Product C.
Consumer 3: Product B is *in fact better* than Product A, and Product A is better (or *almost better)* than Product C.

Considering the consumers’ opinions, we can express their choices as ordinal relations as follows:

Consumer 1: Product B > Product A > Product C
Consumer 2: Product B ≥ Product A ≥ Product C
Consumer 3: Product B > Product A ≥ Product C

**Fig. 1:**
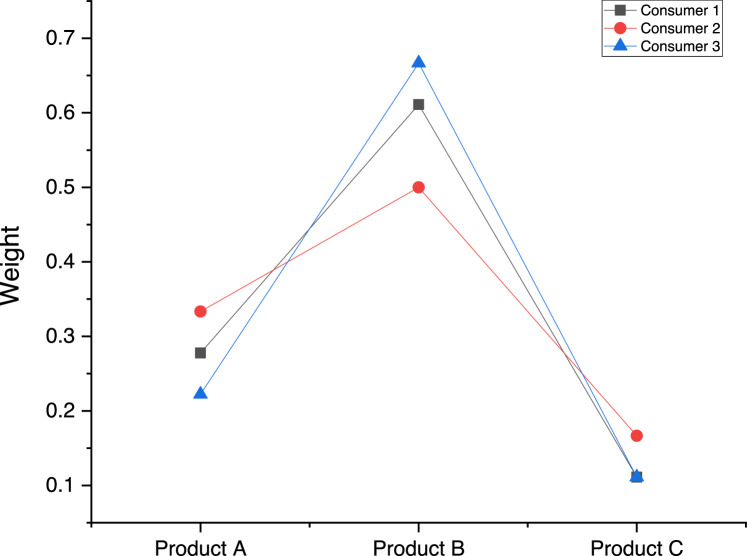
The comparison among the consumers’ choices

**Fig. 2:**
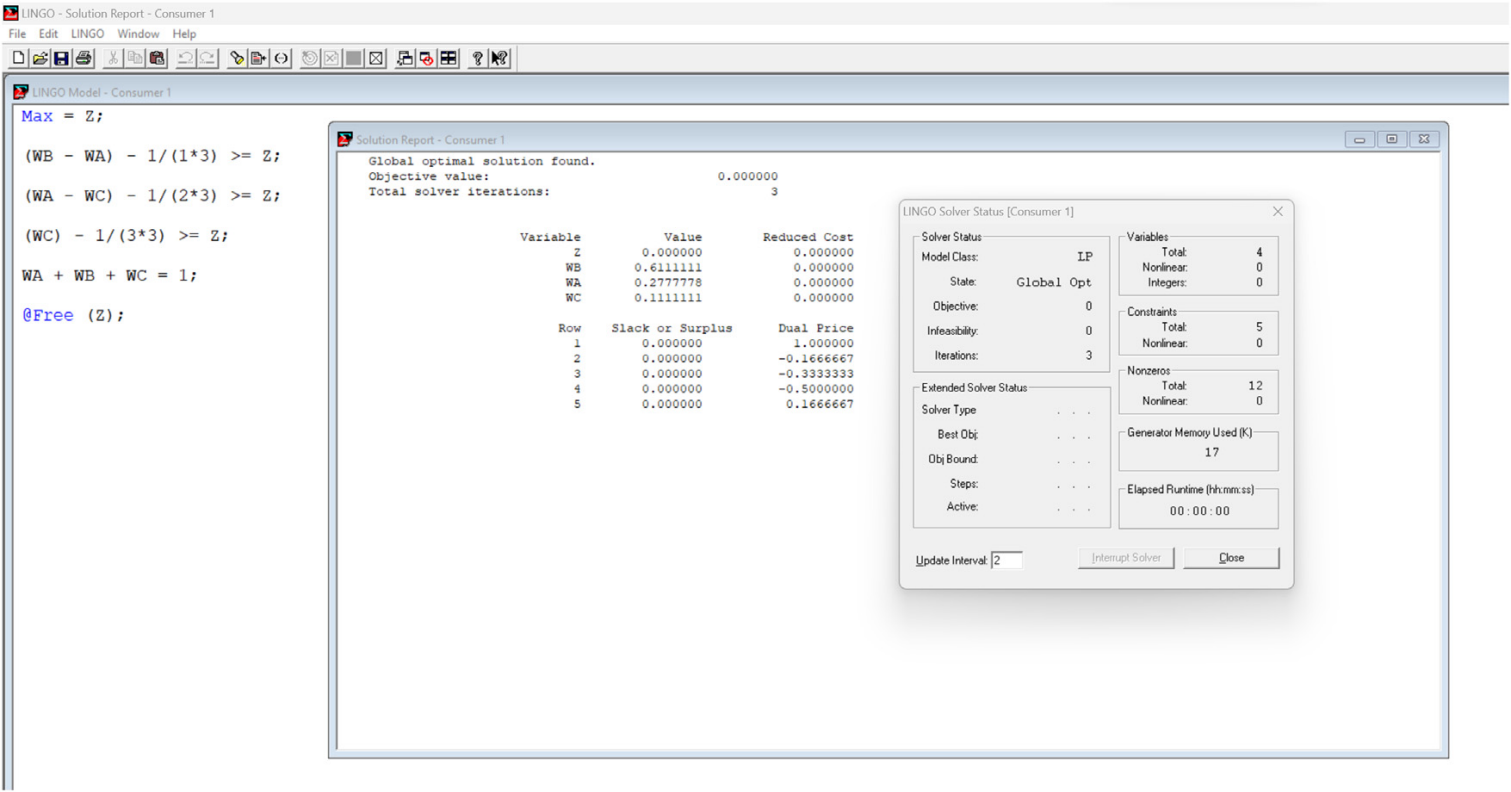
Modeling Consumer 1.

**Fig. 3:**
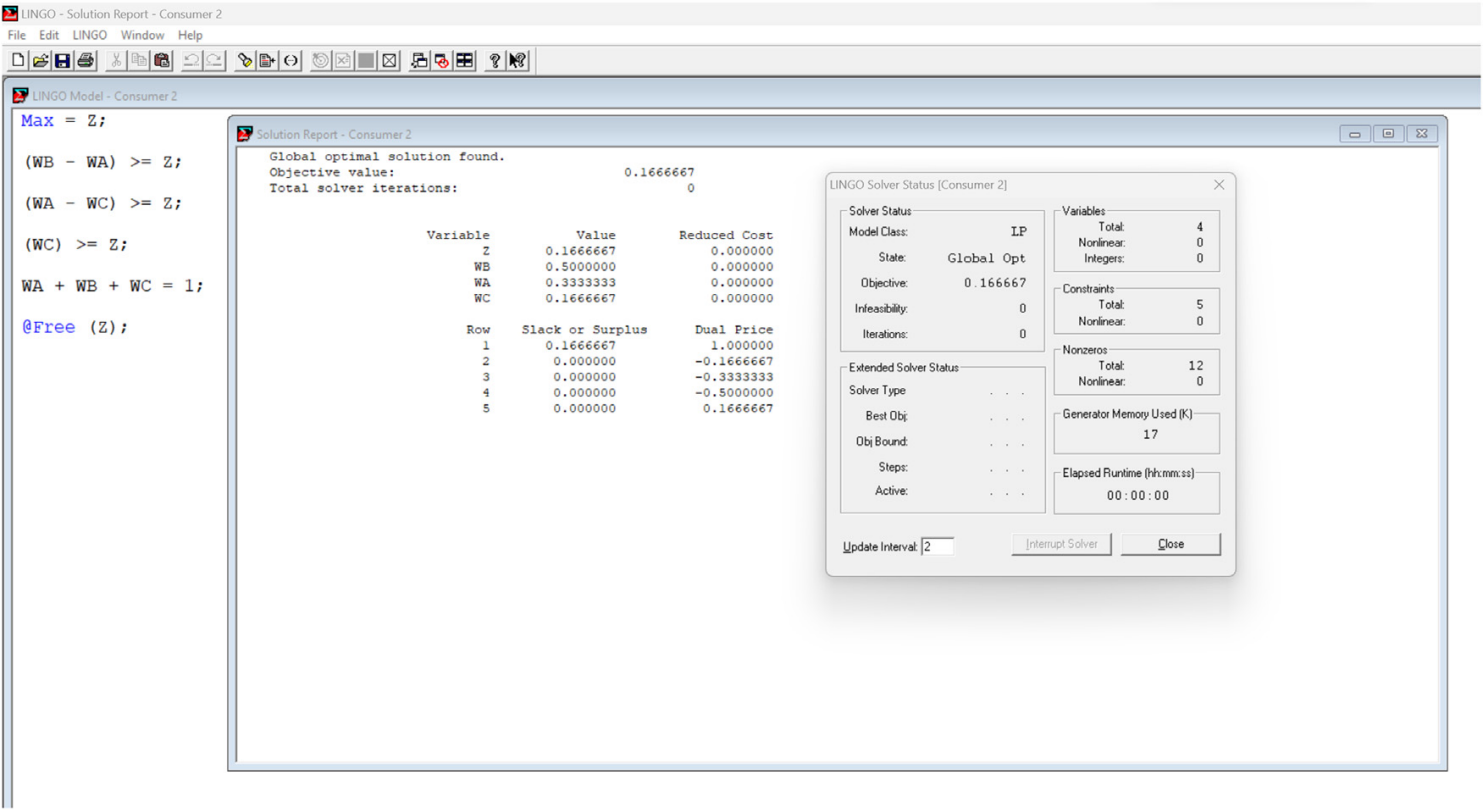
Modeling Consumer 2.

**Fig. 4:**
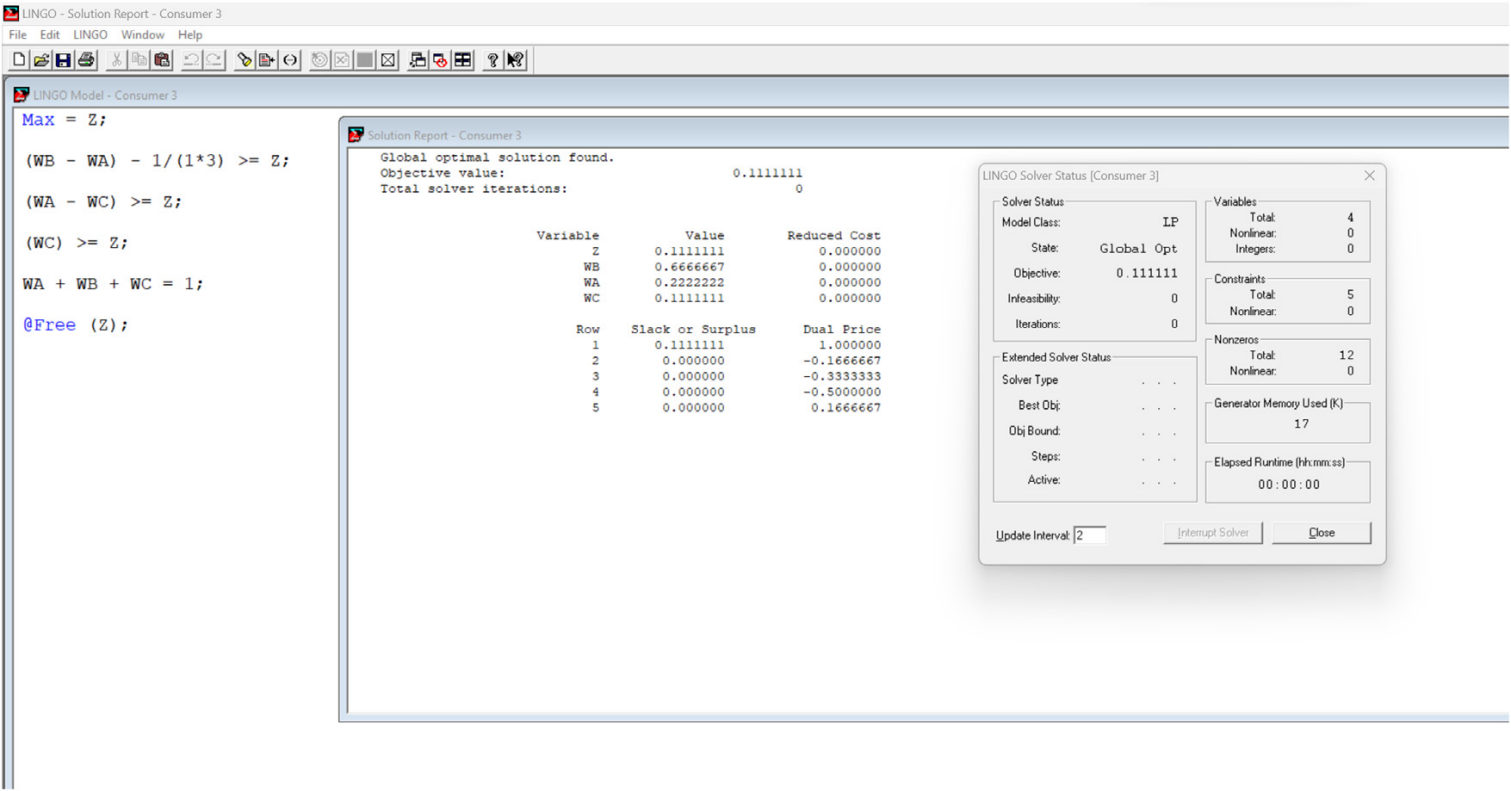
Modeling Consumer 3.

The consumers have been compared in Fig. 1. For modeling Consumer 1, we used Model (13) because all ordinal relations are strict. The implementation of Model (13) in *LINGO 9* software for Consumer 1 is shown in Fig. 2 (Appendix A). For modeling Consumer 2, we used Model (6) because all ordinal relations are weak. The implementation of Model (6) in *LINGO 9* software for Consumer 2 is shown in Fig. 3 (Appendix A). For modeling Consumer 3, we used Model (18) because there are weak and strict ordinal relations simultaneously. The implementation of Model (18) in *LINGO 9* software for Consumer 3 is shown in Fig. 4 (Appendix A).

## Conclusion

The study proves that the classical Ordinal Priority Approach (OPA) model is *strictly ordinal* rather than *weakly ordinal*. Thus, it offers an alternative formulation of the OPA through the development of the OPA with strict ordinal relations (OPA-S). Also, the OPA with weak ordinal relations (OPA-W) is proposed. It also proves the OPA is more robust than the OPA-S. However, both OPA-S and OPA-W have unique applications, demonstrated through a real-world problem. In the future, the hybrid weak-strict model can be applied to various problems to model the experts’ choices. Also, it can be extended for uncertain situations to model real-life problems more precisely.

## Ethics statements

Authors declare to comply with the Journal ethical guidelines.

## CRediT authorship contribution statement

**Amin Mahmoudi:** Conceptualization, Methodology, Software, Validation, Writing – original draft. **Saad Ahmed Javed:** Validation, Writing – original draft.

## Declaration of Competing Interest

The authors declare that they have no known competing financial interests or personal relationships that could have appeared to influence the work reported in this paper.

## Data Availability

No data was used for the research described in the article.
